# Partial differential equations modeling of thermal transportation in Casson nanofluid flow with arrhenius activation energy and irreversibility processes

**DOI:** 10.1038/s41598-022-25010-x

**Published:** 2022-11-29

**Authors:** Khalid Fanoukh Al Oweidi, Wasim Jamshed, B. Shankar Goud, Imran Ullah, Usman, Siti Suzilliana Putri Mohamed Isa, Sayed M. El Din, Kamel Guedri, Refed Adnan Jaleel

**Affiliations:** 1 Department of Water Resources Management Engineering, College of Engineering, Al-Qasim Green University, Babylon, Iraq; 2 Department of Mathematics, Capital University of Science and Technology (CUST), Islamabad, 44000 Pakistan; 3 Department of Mathematics, JNTUH University College of Engineering Hyderabad, Kukatpally, Hyderabad, Telangana 500085 India; 4 College of Civil Engineering, National University of Sciences and Technology, Islamabad, 44000 Pakistan; 5 Department of Computer Science, National University of Sciences and Technology, Balochistan Campus (NBC), Quetta, 87300 Pakistan; 6 Institute for Mathematical Research, Universiti Putra Malaysia (UPM), 43400 Serdang, Selangor Darul Ehsan Malaysia; 7 Centre of Foundation Studies for Agricultural Science, Universiti Putra Malaysia (UPM), 43400 Serdang, Selangor Malaysia; 8 Faculty of Engineering, Center of Research, Future University in Egypt, New Cairo, 11835 Egypt; 9 Mechanical Engineering Department, College of Engineering and Islamic Architecture, Umm Al-Qura University, P. O. Box 5555, Makkah, 21955 Saudi Arabia; 10 Department of Information and Communication Enginèering, Al-Nahrain University, Baghdad, Iraq

**Keywords:** Mathematics and computing, Physics

## Abstract

The formation of entropy in a mixed convection Casson nanofluid model with Arhenius activation energy is examined in this paper using magnetohydrodynamics (MHD). The expanding sheet, whose function of sheet velocity is nonlinear, confines the Casson nanofluid. The final equations, which are obtained from the first mathematical formulations, are solved using the MATLAB built-in solver bvp4c. Utilizing similarity conversion, ODEs are converted in their ultimate form. A number of graphs and tabulations are also provided to show the effects of important flow parameters on the results distribution. Slip parameter was shown to increase fluid temperature and decrease entropy formation. On the production of entropy, the Brinkman number and concentration gradient have opposing effects. In the presence of nanoparticles, the Eckert number effect's augmentation of fluid temperature is more significant. Furthermore, a satisfactory agreement is reached when the findings of the current study are compared to those of studies that have been published in the past.

## Introduction

Heat transfer in the field of thermal engineering entails the usage, manufacture, and conversion of heat power among transportable components. The heat transfer approaches included conduction, convection, and radiation. The transmission of chemical compounds occurs in the heat transfer process. Although those approaches have specific characterizations, they surely arise in the same identical system. The heat variation occurs in the system, while most of the heat remains in the fluid for the convection approach. The convective approach transmits some of the thermal to the circulation^1^. In the industrial field, heat addition, subtraction, or elimination should be performed to achieve an excellent operation in that field. In theory, the system of heat dissipated with the aid of using a warm fluid is different from the system of low thermal energy when the heat is acquired with the assistance of using a low-temperature fluid^2^. The implementation of a warmth switch as a method of heat transmission is 99% in the manufacturing industry. The industrial field implements warmth switch fluids from simple designs to complex structures that execute multiple features within the manufacturing method. A high number of industries that implement the warmth switch are reported since it has various designs appropriate to those industries' requirements^3^. For example, the thermal power system's performance is assisted by using the heat exchanger, where the heat exchanger acts as a warmth switch. Miniaturization of heat exchangers greatly turns them more compact and green. Meanwhile, a micro-channel heat sink is widely used in electronic cooling and also it completely green heat exchanger^4^.

New energy sources have been developed as a result of contemporary research in nanotechnology to improve the efficiency of sophisticated thermal systems. Nanofluids are formed by submerging particles in an elemental liquid, where the size of particles is nanometers. Various types of base fluid and nanoparticles have been used to form nanofluids as a heat transfer medium for different processes. Water, motor oils, and ethylene glycol have become the top selection as a base fluid in a nanofluid. Water is not the best selection because it has low thermal conductivity whether it is a renewable source. Besides, motor oils and ethylene glycol have high viscosity but are toxic to the environment^5^. A mixture of ethylene glycol or water with nanoparticles is used as a car coolant for engine performance. High-performance computers also employ electronic cooling technology in a microprocessor circuit to reach a maximum power of 100,300 W/cm^2^ ^6^. Meanwhile, natural convection occurred in the flow of nanofluid which is observed by the thermal conductivity and viscosity and is used as a working fluid to transfer heat^7^.

Buongiorno, who proposed a non-homogeneous version, diagnosed seven elements that might contribute to the improvement of warmth switch to Nanofluid; however, by and large of them, the Brownian motion and thermophoresis had been determined to be the maximum contributing elements^8^. The outcomes of viscous heat, thermal radiation, and the decided situations of the higher temperature variety also are considered. A concerted attempt has been made to the modified version of the Buongiorno mathematical model with the presence of gyrotactic microorganisms, thermophoresis, and Brownian motion. Subsequently, the Buongiorno changed version is used for a bioconvective float of gyrotactic microorganisms^9^. The Buongiorno version is primarily based totally on thermo diffusion and random motion of nanoparticles. This version became utilized by numerous researchers to examine the dynamics of nanofluid flow over a flat plate, which analyze the variation of the flow, and the transmission of heat and mass. The Buongiorno's Model is selected by Puneeth et al.^10^ to evaluate the magnetic radiating nanofluid flow throughout the boundary with a cone, considering chemical reactions. The report on a fluid retention of alumina and titania debris close to a horizontal extended sheet is published by Rana et al.^11^. The risky shipping of hybrid Nanofluid over long distances using the Buongiorno's model is tested by Ali et al.^12^, and they observed that the velocity is increased. Meanwhile, the thermophoretic placement hurries the Reynolds range and the temperature distinction among air and wall. Brownian motion is defined as the random movement of the debris suspended in the fluid^13^. The pioneer document on the Brownian motion was reported by Jan Ingenhousz in 1785, regarding the coal dirt inside alcohol. Later, Albert Einstein derive a mathematics formula to define Brownian motion. Garg and Jayaraj^14^ recently defined the Brownian motion of aerosol debris in crossflow with cylindrical geometry.

The non-Newtonian fluid phenomena have a widespread position in sustainable electricity and renewable structures of cutting-edge trends. The human blood has a rheological property of the Casson fluid, which is one type of non-Newtonian fluids. The mathematical analysis of the Casson fluid have been reported^15–38^, due to the external impacts of slip conditions and Joule heating^15,16^, convective boundary conditions^17,18^, radiation^19,20^, chemical reaction^20,21^, magnetic field^22–29^, porous boundary sheet^30,31^, viscous dissipation^32,33^, heat generation and heat sink^34,35^, and various thermal conductivity^36,37^. Mixed nanofluids are novel nanofluids organized via way of forming extraordinary nanoparticles both in combination or in a composite form. The impetus for the training of composite nanofluids is the non-stop development of heat transfer with the advanced thermal conductivity of those nanofluids. Among all the hybrid nanofluids tested, the waft characteristics and heat transfer traits of the CNT/Fe_3_O_4_ nanofluid are extensively analyzed^38^. Akbari^39^ measured via way of means of viscosity of ethylene glycol/MgO-MWCNT hybrid nanofluid at quantity ratios of nanoparticles starting from zero to 1% inside a temperature variety of 30 to 60° C. The CNT / Fe_3_O_4_ nanoparticles in nanofluid are used as a cooler in a small channel temperature changer, and its houses are numerically tested. Waqas et al.^40^ explored the effect of thermal radiation in hybrid nanofluid for Powell-Erying model. The heat transfer enhancement in the mixed convection flow of hybrid nanofluid with temperature jump was reported by Khalid et al.^41^.

Activation energy executes a critical task in convection boundary layer flows, with the presence of heat and mass transmission. For instance, activation energy is the activation of electricity that occurs in the oil and geothermal reservoirs. Several studies regarding to the activation electricity are reported with the various impacts and model/situation: three-dimensional model with slip and binary chemical reactions^42^, peristaltic flow in a curvy channel with diverse thermal conductivity^43^, the Nield model of a stretching sheet with the nonlinear radiative heat flux^44^, and the model of bio-convective Sisko fluid version, which consists of microorganisms. Other applicable research on the activation of electricity with the gyrotactic microorganisms is listed in references^45–47^.

Entropy is a systematic idea and measurable cloth regularly related to a nation of distraction, disorder, or uncertainty. Rudolf Clausius (1822–1888) is the founding father of the idea of entropy. Austrian physicist Ludwig Boltzmann defined entropy as a degree of the quantity of feasible microscopic structures or areas of man or woman atoms and molecules of a device compliant with the macroscopic device^48^. Entropy technology evaluation is a useful device for enhancing the overall performance of thermal structures. It is thought that adding nanoparticles to simple fluids can contribute to the total technology of entropy^49^. Therefore, using Nanofluids in thermal structures reduces device temperature, and in the long run, the warmth switch contribution to the overall quantity of entropy manufacturing decreases, while nanoparticles introduced to simple fluid boom the viscosity of the lively fluid main to decreased device pressure. Manjunath and Kaushik^50^ reviewed research primarily based totally on second-regulation evaluation implemented to heaters. Subsequently, Waqas et al.^51^ develop a model of entropy technology for Casson nanofluid in the presence of convective boundary conditions. On the other hand, Farooq et al.^52^ investigated the impact of nonlinear thermal radiation in the nanofluid with entropy generation. Regarding the technology of entropy withinside the flow of nanofluid / hybrid Nano fluid, the best evaluations have been performed by Mahian et al.^53^. The improvement and usage of Nano fluids has widely implemented in customer products, Nanomedicine, electricity conversion, and microsystem cooling. Of specific hobby is using Nano fluid go with the drift to enhance convection warmness switch to obtain quicker cooling of excessive bendy devices. However, if you want to nicely broaden such thermal engineer’s structures in terms of layout and overall performance, now no longer does the best warmness switch need to be superior; entropy technology needs to be decreased^54^.

Based on literature review, it has been clear that no attention is paid to study the mixed convection flow of Casson nanofluid with entropy generation and activation energy. In present analysis, we presented the graphical results of mixed convection flow of Casson nanofluid in the presence of entropy generation. The MATLAB software's built-in bvp4c approach is used to generate the mathematical results for the fluid flow, temperature gradient, and entropy production. These discoveries might help engineers create better cooling methods for applications like nuclear power plants, heat transfers, photovoltaic collectors, and electrical device refrigeration. The current analysis has applications in plasma investigations, crystal growth, atmospheric fallout, geothermal energy recovery, nuclear reactor cooling, paint spraying, etc. High temperatures are necessary for some electronics to operate effectively. Thermal radiation is used to determine the thermal impact of huge engines, heat exchangers, power plants, and rack nozzles.

## Mathematical formulation

**Figure 1:**
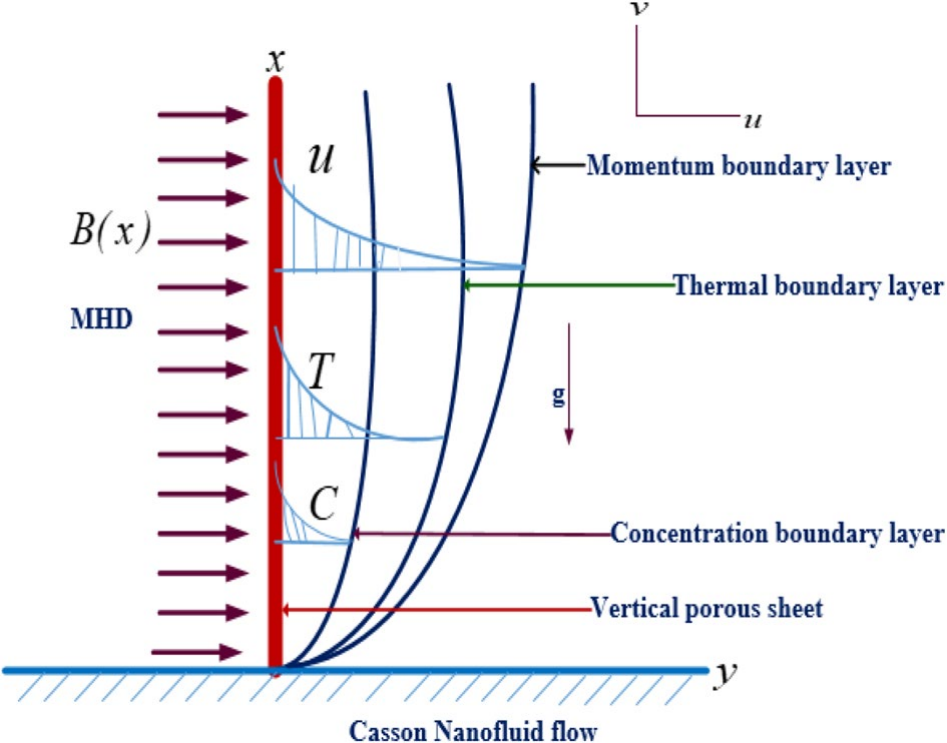
Geometric flowing diagram.

The fluid is designed to move across an extended surface and has a two-dimensional flow. The effects of thermal radiation, entropy formation, and the slip phenomenon are investigated. In the current study, the coordinate system, as well as the physical and graphic modeling, is also described. The representation of the nanofluid model is depicted in Fig. 1, and the characteristics of the problem are as follow:

- The mixed convective flow of Casson nanofluid.
- The nanofluid is bounded by a slipped and convective sheet.
- The sheet is stretched nonlinearly and it is expressed as $u_{w} (x) = ax^{m}$, where $a$ is constant.
- The sheet and the flow direction are placed along the *x-* and *y-*axes, respectively.
- The temperature and concentration at free stream are $T_{\infty }$ and $C_{\infty }$, respectively.

The rheological equation of state for an isotropic and incompressible flow of Casson fluid is given by:

1 $$\tau _{{ij}} = \left\{ {\begin{array}{*{20}c} {2\left( {\mu _{B} + \frac{{p_{y} }}{{\sqrt {2\pi } }}} \right)e_{{ij}} ,\;\pi > \pi _{c} } \\ {2\left( {\mu _{B} + \frac{{p_{y} }}{{\sqrt {2\pi _{c} } }}} \right)e_{{ij}} ,\;\pi < \pi _{c} } \\ \end{array} } \right.$$

In the above equation, $\pi$ is the product of the deformation rate component and itself; i.e., $\pi ={e}_{ij}{e}_{ij}$ and ${e}_{ij}$ is the $(i,j)th$ component of the deformation rate. ${\pi }_{c}$ is the critical value of this product based on the non-Newtonian model. ${\mu }_{B}$ is the plastic dynamic viscosity of the non-Newtonian fluid, and ${p}_{y}$ signifies the yield stress of the fluid.

The controlling equations are given below^21–23^:

Continuity Equation

2 $$u_{x} + v_{y} = 0$$

Momentum Equation

3 $$\begin{gathered} u\,u_{x} + v\,u_{y} = \left( {{{\mu_{f} } \mathord{\left/ {\vphantom {{\mu_{f} } {\rho_{f} }}} \right. \kern-\nulldelimiterspace} {\rho_{f} }}} \right)\,u_{yy} + \left( {1 + {1 \mathord{\left/ {\vphantom {1 \beta }} \right. \kern-\nulldelimiterspace} \beta }} \right)\,u_{yy} - \left( {{{\sigma B^{2} (x)} \mathord{\left/ {\vphantom {{\sigma B^{2} (x)} {\rho_{f} }}} \right. \kern-\nulldelimiterspace} {\rho_{f} }}} \right)\,u \hfill \\ \, + \left[ {\left( {1 - C_{\infty } } \right)\left( {{{\rho_{{f_{\infty } }} } \mathord{\left/ {\vphantom {{\rho_{{f_{\infty } }} } {\rho_{f} }}} \right. \kern-\nulldelimiterspace} {\rho_{f} }}} \right)\beta_{T} \left( {T - T_{\infty } } \right) - \left( {{{\left( {\rho_{p} - \rho_{{f_{\infty } }} } \right)} \mathord{\left/ {\vphantom {{\left( {\rho_{p} - \rho_{{f_{\infty } }} } \right)} {\rho_{f} }}} \right. \kern-\nulldelimiterspace} {\rho_{f} }}} \right)(C - C_{\infty } )} \right]g, \hfill \\ \end{gathered}$$

Energy Equation

4 $$\begin{gathered} u\,T_{x} + v\,T_{y} = \alpha_{f} \,T_{yy} + \tau \left[ {D_{B} \,C_{y} \,T_{y} + \left( {{{D_{T} } \mathord{\left/ {\vphantom {{D_{T} } {T_{\infty } }}} \right. \kern-\nulldelimiterspace} {T_{\infty } }}} \right)\,T_{y}^{2} \,} \right] - \left( {{1 \mathord{\left/ {\vphantom {1 {\left( {\rho c} \right)_{f} }}} \right. \kern-\nulldelimiterspace} {\left( {\rho c} \right)_{f} }}} \right)\frac{1}{{\left( {\rho c} \right)_{f} }}\,\left( {q_{r} } \right)_{y} \hfill \\ \, + \left( {{{\mu_{f} } \mathord{\left/ {\vphantom {{\mu_{f} } {\left( {\rho c} \right)_{f} }}} \right. \kern-\nulldelimiterspace} {\left( {\rho c} \right)_{f} }}} \right)\left( {1 + \left( {{1 \mathord{\left/ {\vphantom {1 \beta }} \right. \kern-\nulldelimiterspace} \beta }} \right)} \right)\left( {\frac{\partial u}{{\partial y}}} \right)^{2} \,u_{y}^{2} + \left( {{{\sigma B_{0}^{2} } \mathord{\left/ {\vphantom {{\sigma B_{0}^{2} } {\left( {\rho c} \right)_{f} }}} \right. \kern-\nulldelimiterspace} {\left( {\rho c} \right)_{f} }}} \right)\,u^{2} \hfill \\ \, + \left( {{Q \mathord{\left/ {\vphantom {Q {\left( {\rho c} \right)_{f} }}} \right. \kern-\nulldelimiterspace} {\left( {\rho c} \right)_{f} }}} \right)\left( {T - T_{\infty } } \right) \hfill \\ \end{gathered}$$

Concentration Equation

5 $$u\,C_{x} + v\,C_{y} = D_{B} \,C_{yy} + \left( {{{D_{T} } \mathord{\left/ {\vphantom {{D_{T} } {T_{\infty } }}} \right. \kern-\nulldelimiterspace} {T_{\infty } }}} \right)\,T_{yy} - k_{r}^{2} \left( {C - C_{\infty } } \right)\left( {{T \mathord{\left/ {\vphantom {T {T_{\infty } }}} \right. \kern-\nulldelimiterspace} {T_{\infty } }}} \right)^{n} e^{{\left( {{{ - E_{a} } \mathord{\left/ {\vphantom {{ - E_{a} } {kT}}} \right. \kern-\nulldelimiterspace} {kT}}} \right)}}$$

where the subscripts *x* and *y* are the differentiation in terms of *x* and *y*, respectively. Besides, velocity in vector *x* and *y* are indicated by $$u$$ and $$v$$ respectively. Meanwhile, another symbols such as $$\mu_{f}$$, $$\sigma ,$$ $$\rho_{f} ,$$ $$g,$$ $$\beta_{T} ,$$ $$\alpha_{f} = {k \mathord{\left/ {\vphantom {k {\left( {\rho c} \right)_{f} }}} \right. \kern-\nulldelimiterspace} {\left( {\rho c} \right)_{f} }},$$ $$k,$$ $$\left( {\rho c} \right)_{f} ,$$$$\tau = {{\left( {\rho c} \right)_{p} } \mathord{\left/ {\vphantom {{\left( {\rho c} \right)_{p} } {\left( {\rho c} \right)_{f} ,}}} \right. \kern-\nulldelimiterspace} {\left( {\rho c} \right)_{f} ,}}$$ $$\left( {\rho c} \right)_{p} ,$$ $$D_{B} ,$$ $$D_{T} ,$$ $$q_{r} ,$$ $$Q,$$ and $$k_{r}^{2}$$ are defined as follow: dynamic viscosity of the fluid, electrical conductivity, fluid density, gravitational acceleration, volumetric coefficient of thermal expansion, thermal diffusivity of the fluid, thermal conductivity of the fluid, heat capacity of the fluid, ratio of heat capacities, effective heat capacity of nanoparticles material, Brownian diffusion coefficient, thermophoretic diffusion coefficient, radiative heat flux, heat generation/absorption coefficient, and rate of a chemical reaction. Specifically, the radiative heat flux $$q_{r}$$ is derived as $$q_{r} = \left( {{{ - 4\sigma^{ * } } \mathord{\left/ {\vphantom {{ - 4\sigma^{ * } } {3k_{1}^{*} }}} \right. \kern-\nulldelimiterspace} {3k_{1}^{*} }}} \right)\;\left[ {\left( {4T_{\infty }^{3} T - 3T_{\infty }^{4} } \right)_{y} } \right]^{4}$$ ^52^, where Stefan-Boltzmann constant and mean absorption coefficient are denoted by $$\sigma^{ * }$$ and $$k_{1}^{*}$$, respectively.

The restricted conditions at the distance $y = 0$ and $y \to \infty$ are listed as below, where $N_{1} = N_{0} x^{{ - \left( {{{m - 1} \mathord{\left/ {\vphantom {{m - 1} 2}} \right. \kern-\nulldelimiterspace} 2}} \right)}}$ is the velocity slip, $h_{f} = h_{0} x^{{\left( {{{m - 1} \mathord{\left/ {\vphantom {{m - 1} 2}} \right. \kern-\nulldelimiterspace} 2}} \right)}} ,$ is the convective heat transmission, and $h_{s} = h_{0} x^{{\left( {{{m - 1} \mathord{\left/ {\vphantom {{m - 1} 2}} \right. \kern-\nulldelimiterspace} 2}} \right)}}$ is the convective mass transmission.

Boundary Conditions

6 $$u = u_{w} + N_{1} \nu \,u_{y} \,,v = V_{w} ,k\,T_{y} = - h_{f} \left( {T_{f} - T} \right),D_{B} \,C_{y} = - h_{s} \left( {C_{w} - C} \right){\text{at }}y = 0,$$

7 $$u \to 0,\quad T \to T_{\infty } { ,}\quad C \to C_{\infty } \quad {\text{as}}\;\;y \to \infty {.}$$

## Solution methodology

The stream function $$\psi$$, a similarity variable $$\eta$$, and the conversion for temperature $$\theta$$ and concentration $$\phi$$ (where $$f,$$$$\theta$$ and $$\phi$$ are the function of $$\eta$$) are expressed as

8 $$\eta = \sqrt {\frac{{(m + 1)ax^{m} }}{2\nu x}} y,\quad \psi = \sqrt {\frac{{2\nu ax^{m + 1} }}{m + 1}} f,\quad \theta = \frac{{T - T_{\infty } }}{{T_{f} - T_{\infty } }}\,,\quad \phi = \frac{{C - C_{\infty } }}{{C_{w} - C_{\infty } }}$$

By using Eq. 7, the Eqs. (2–6) will become

9 $$\left( {1 + \frac{1}{\beta }} \right)f_{\eta \eta \eta } + ff_{\eta \eta } \, - \frac{2m}{{m + 1}}f_{\eta }^{2} - \frac{2}{m + 1}Mf_{\eta } + \lambda \left( {\theta + N\phi } \right) = 0,$$

10 $$\begin{gathered} \hfill \frac{1}{\Pr }\left( {1 + \frac{4}{3}R_{d} } \right)\theta_{\eta \eta } \, + f\theta_{\eta } + N_{b} \phi_{\eta } \theta_{\eta } + N_{t} \theta_{\eta }^{2} + (1 + \frac{1}{\beta })Ecf_{\eta \eta }^{2} \\ \hfill + MEcf_{\eta }^{2} + \varepsilon \theta = 0 \\ \end{gathered}$$

11 $$\frac{1}{Le}\phi_{\eta \eta } + f\phi_{\eta } + \frac{{N_{t} }}{{N_{b} }}\theta_{\eta \eta } - \left( {\frac{2}{m + 1}} \right)k_{1} (1 + \alpha_{1} \theta )^{n} \phi \exp (\frac{ - E}{{1 + \alpha_{1} \theta }}) = 0$$

where the subscript $\eta$ denotes the differentiation in this symbol.

The transformed controlling conditions from Eqs. 6–7 are:

12 $$f(\eta ) = 0,f_{\eta } = 1 + \sqrt {\frac{m + 1}{2}} \delta f_{\eta \eta } ,\,\theta_{\eta } = - \sqrt {\frac{2}{m + 1}} Bi_{1} \left[ {1 - \theta } \right],\phi_{\eta } = - \sqrt {\frac{2}{m + 1}} Bi_{2} \left[ {1 - \phi } \right],{\text{ at }}\eta = 0,$$

13 $$\;f_{\eta } = 0,\,\quad \quad \theta = 0,\quad \quad \phi = 0 \, \quad {\text{ as}}\;\;\eta \to \infty$$

From Eqs. 9–13, $M$, $\lambda$, $N$, $\Pr$, $R_{d}$, $N_{t}$, $N_{b}$, $Ec$, $\varepsilon$ ($\varepsilon > 0$ is for heat generation and $\varepsilon < 0$ denotes heat absorption), $Le$, $k_{1}$, $\alpha_{1}$, $E$, $\delta$ and $Bi_{1}$, $Bi_{2}$ are the magnetic parameter, mixed convection, buoyancy forces ratio, Prandtl number, radiation parameter, thermophoresis parameter, Brownian motion parameter, Eckert number, heat generation/absorption parameter, Lewis number, reaction rate, temperature gradient, activation energy parameter, slip parameter and Biot numbers, and are defined as

14 $$\begin{gathered} M = \frac{{\sigma B_{0}^{2} }}{{a\rho_{f} }}\;\,,\;\lambda = \frac{Gr}{{{\text{Re}}_{x}^{2} }},\;N = \frac{{\left( {\rho_{p} - \rho_{{f_{\infty } }} } \right)\left( {C_{w} - C_{{_{\infty } }} } \right)}}{{\left( {1 - C_{\infty } } \right)\rho_{{f_{\infty } }} \beta_{T} \left( {T_{f} - T_{\infty } } \right)}},\;\mathop {\Pr }\limits = \frac{{\nu_{f} }}{{\alpha_{f} }},\;R_{d} = \frac{{4\sigma^{ * } T_{\infty }^{3} }}{{kk_{1}^{*} }}, \hfill \\ N_{t} = \frac{{\tau D_{T} (T_{f} - T_{\infty } )}}{\nu },\;N_{b} = \frac{{\tau D_{B} (C_{w} - C_{\infty } )}}{\nu },\;Ec = \frac{{u_{w}^{2} }}{{c_{f} \left( {T_{f} - T_{\infty } } \right)}},\;\varepsilon = \frac{Q}{{\left( {\rho c} \right)_{f} a}},\;Le = \frac{\nu }{{D_{B} }},\;k_{1} = \frac{{k_{r}^{2} }}{a}, \hfill \\ \alpha_{1} = \frac{{T_{f} - T_{\infty } }}{{T_{{_{\infty } }} }},\;E = \frac{{ - E_{a} }}{{kT_{f} }},\;\delta = N_{0} \sqrt {\frac{a}{\nu }} ,\;Gr = \frac{{\left( {1 - C_{\infty } } \right)\left( {{\raise0.7ex\hbox{${\rho_{{f_{\infty } }} }$} \!\mathord{\left/ {\vphantom {{\rho_{{f_{\infty } }} } {\rho_{f} }}}\right.\kern-\nulldelimiterspace} \!\lower0.7ex\hbox{${\rho_{f} }$}}} \right)g\beta_{T} (T_{\infty } - T_{m} )x^{3} }}{{\nu^{2} }} \hfill \\ \end{gathered}$$

The equations of the wall skin friction, wall heat flux, and wall mass flux are:

15 $$\tau_{w} = \mu \left( {1 + \frac{1}{\beta }} \right)\,\left( {u_{y} } \right)^{2}_{y = 0} ,\,\;q_{w} = - \left( {\left( {\alpha_{f} + \frac{{16\sigma^{ * } T_{\infty }^{3} }}{{3\rho c_{p} k_{1} {}^{*}}}} \right)\,T_{y} } \right)_{y = 0} ,q_{s} = - D_{B} \left( {C_{y} } \right)_{y = 0}$$

The dimensionless skin friction coefficient $Cf_{x} = \frac{{2\tau_{w} }}{{\rho_{f} u_{w}^{2} }}$, the local Nusselt number $Nu_{x} = \frac{{xq_{w} }}{{\alpha_{f} (T_{f} - T_{\infty } )}}$ , and local Sherwood number $Sh_{x} = \frac{{xq_{s} }}{{D_{B} (C_{w} - C_{\infty } )}}$ can be derived from Eq. 14, and finally we obtain

16 $$\begin{gathered} \left( {Re_{x} } \right)^{1/2} Cf_{x} = \left( {\frac{m + 1}{2}} \right)\left( {1 + \frac{1}{\beta }} \right)f_{\eta \eta } (0),\,\quad \hfill \\ \left( {Re_{x} } \right)^{ - 1/2} Nu_{x} = - \left( {\frac{m + 1}{2}} \right)\left( {1 + \frac{4}{3}R_{d} } \right)\theta_{\eta } (0), \hfill \\ (Re)^{ - 1/2} Sh_{x} = - \left( {\frac{m + 1}{2}} \right)\phi_{\eta } (0)\, \hfill \\ \end{gathered}$$

where ${\text{Re}}_{x} = \frac{{ax^{m - 1} }}{\nu }$ is the local Reynold number.

## Entropy generation and modeling

The entropy generation is mathematically expressed as

17 $$S_{G} = \frac{k}{{T_{\infty }^{2} }}(1 + \frac{{16\sigma^{*} T_{\infty }^{3} }}{{3kk^{*} }})\left( {T_{y} } \right)^{2} + \frac{{\sigma B^{2} (x)}}{{T_{\infty } }}u^{2} + \frac{{\mu_{f} }}{{T_{\infty } }}\left( {1 + \frac{1}{\beta }} \right)\left( {u_{y} } \right)^{2} + \frac{{RD_{B} }}{{T_{\infty } }}C_{y} T_{y} + \frac{{RD_{B} }}{{C_{\infty } }}\left( {C_{y} } \right)^{2}$$

Which, after simplification, gives the form

18 $$\begin{gathered} N_{G} = \left( {1 + \frac{4}{3}R_{d} } \right)\left( {\frac{m + 1}{2}} \right)\theta_{\eta }^{2} \alpha_{1} \, + M\frac{Br}{{\alpha_{1} \,}}f_{\eta }^{2} + \left( {\frac{m + 1}{2}} \right)\left( {1 + \frac{1}{\beta }} \right)\frac{Br}{{\alpha_{1} \,}}f_{\eta \eta }^{2} + \left( {\frac{m + 1}{2}} \right)\frac{{\chi \lambda_{1} }}{{\alpha_{1} }}\phi_{\eta } \theta_{\eta } \hfill \\ + \left( {\frac{m + 1}{2}} \right)\left( {\frac{\chi }{{\alpha_{1} }}} \right)^{2} \lambda_{1} \phi_{\eta }^{2} \hfill \\ \end{gathered}$$

Here.

$$N_{G} = \frac{{\nu S_{G} T_{\infty }^{2} }}{{ak\left( {T_{f} - T_{\infty } } \right)^{2} }}x^{1 - m}$$, $$Br = \frac{{\mu a^{2} x^{2m} }}{{k\left( {T_{f} - T_{\infty } } \right)}}$$, $$\chi = \frac{{(C_{w} - C_{\infty } )}}{{C_{\infty } }}$$, $$\lambda_{1} = \frac{{RD_{B} C_{\infty } }}{k}$$

Where $N_{G}$, $Br$, $\chi$ and $\lambda_{1}$ are the rate of entropy optimization rate, Brinkman number, concentration gradient and diffusive variable respectively.

## Numerical procedure

The appropriate numerical method with accurate convergence must be used for the equations system. The numerical findings are obtained using a bvp4c MATLAB method^21,51,52^. Compared to other numerical approaches, the bvp4c methodology is more adaptable and allows for more precise control of approach criteria. The following are the components of the computing scheme:

Using an appropriate substitution such below:

19 $$y\left( 1 \right) = f,\;y\left( 2 \right) = f_{\eta } ,\;f\left( 3 \right) = f_{\eta \eta } ,\;y\left( 4 \right) = \theta ,\;y\left( 5 \right) = \theta_{\eta } ,\;y\left( 6 \right) = \emptyset ,\;y\left( 7 \right) = \emptyset_{\eta }$$

The first-order system of equation is obtained:

20 $$\left( {\begin{array}{*{20}c} {y_{\eta } \left( 1 \right)} \\ {y_{\eta } \left( 2 \right)} \\ {y_{\eta } \left( 3 \right)} \\ {y_{\eta } \left( 4 \right)} \\ {y_{\eta } \left( 5 \right)} \\ {y_{\eta } \left( 6 \right)} \\ {y_{\eta } \left( 7 \right)} \\ \end{array} } \right) = \left( {\begin{array}{*{20}c} {y\left( 2 \right)} \\ {y\left( 3 \right)} \\ {\left( {\frac{ - 1}{{1 + \frac{1}{\beta }}}} \right)\left( {\begin{array}{*{20}c} {y\left( 1 \right)*y\left( 3 \right) - \frac{2m}{{m + 1}}\left( {y\left( 2 \right)} \right)^{2} - \frac{2}{m + 1}*M*y\left( 2 \right)} \\ { + \lambda *\left( {y\left( 4 \right) + N*y\left( 6 \right)} \right)} \\ \end{array} } \right)} \\ {f\left( 5 \right)} \\ {\left( {\frac{ - 1}{{1 + \frac{4R}{3}}}} \right)\left( {Pr*\left( {\begin{array}{*{20}c} {\left( {y\left( 1 \right)*y\left( 5 \right) + Nb*y\left( 5 \right)*y\left( 7 \right) + Nt*y\left( 5 \right)^{2} } \right)} \\ { + \left( {1 + \frac{1}{\beta }} \right)*Ec*\left( {y\left( 3 \right)} \right)^{2} + M*Ec*\left( {y\left( 2 \right)} \right)^{2} + \varepsilon *y\left( 4 \right)} \\ \end{array} } \right)} \right)} \\ {y\left( 7 \right)} \\ {Le*\left( {\begin{array}{*{20}c} {y\left( 1 \right)*y\left( 7 \right) - \frac{2}{m + 1}*k_{1} *\left( {1 + \alpha_{1} *y\left( 4 \right)} \right)^{2} *} \\ {y\left( 6 \right)*\exp \left( { - \frac{E}{{\left( {1 + \alpha_{1} *y\left( 4 \right)} \right)}}} \right)} \\ \end{array} } \right) - Le*\frac{{N_{t} }}{{N_{b} }}*y_{\eta } \left( 5 \right)} \\ \end{array} } \right)$$

The changed initial and boundary constraints as specified in the following:

21 $$\left( {\begin{array}{*{20}c} {y_{a} \left( 1 \right)} \\ {y_{a} \left( 2 \right)} \\ {y_{a} \left( 5 \right)} \\ {y_{a} \left( 7 \right)} \\ {y_{b} \left( 2 \right)} \\ {y_{b} \left( 4 \right)} \\ {y_{b} \left( 6 \right)} \\ \end{array} } \right) = \left( {\begin{array}{*{20}c} 0 \\ {1 + \sqrt {\frac{m + 1}{2}} *\delta *y_{a} \left( 3 \right)} \\ { - \sqrt {\frac{2}{m + 1}} Bi_{1} \left( {1 - y_{a} \left( 4 \right)} \right)} \\ { - \sqrt {\frac{2}{m + 1}} Bi_{2} \left( {1 - y_{a} \left( 6 \right)} \right)} \\ 0 \\ 1 \\ 0 \\ \end{array} } \right).$$

**Figure 2:**
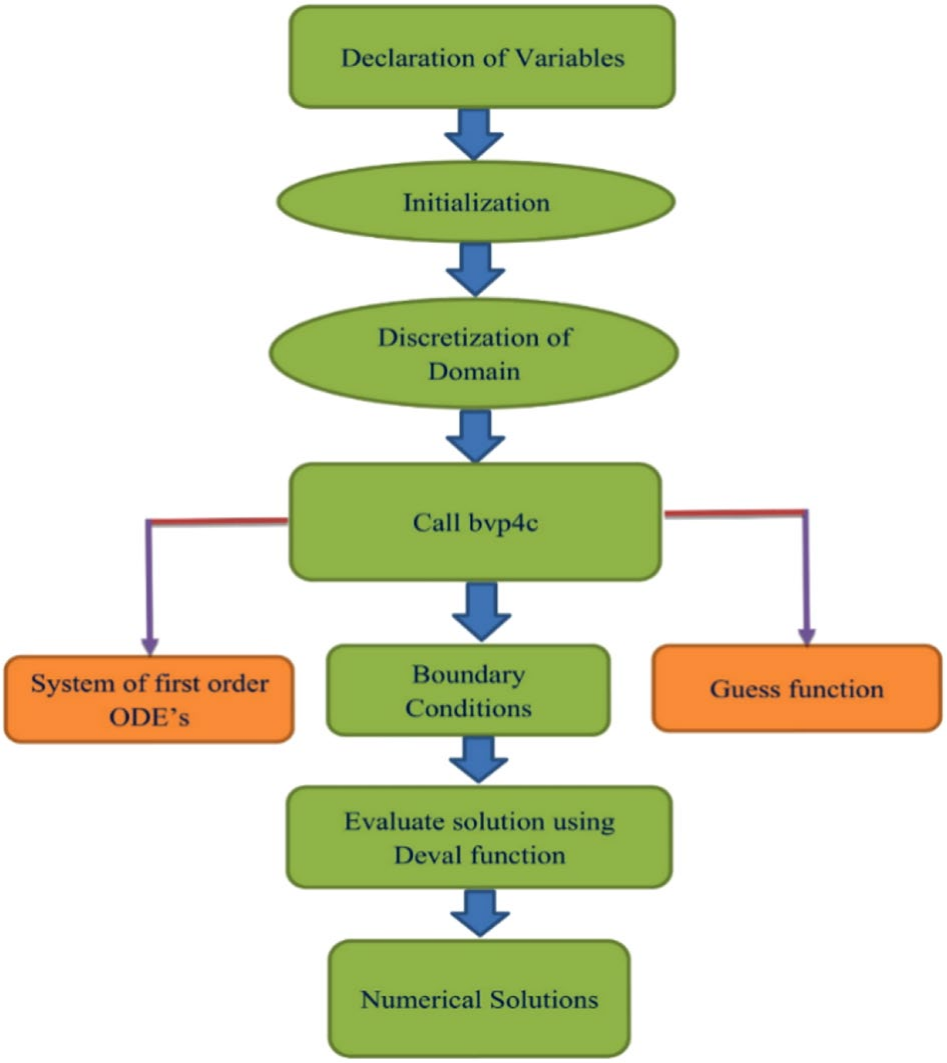
The steps of numerical solutions.

In this stage, we need to select the appropriate finite approximation values of ${\eta }_{\infty }$. As a result, to approximate the values of ${\eta }_{\infty }=10$. The boundary is still set to ${10}^{-4}$ . The value of ${\eta }_{\infty }\to 10$ shows that under this technique, each numerical answer exactly satisfies asymptotic characteristics. A detailed flow diagram has also been included for a better understanding of the current approach bvp4c technique. (see Fig. 2).

## Results and discussion

The outcomes from this model demonstrate the impression of the pertinent parameters profiles of velocity $f^{\prime}\left( \eta \right)$, temperature $\theta \left(\eta \right)$, concentration $\phi \left(\eta \right)$, and entropy generation ${N}_{G}\left(\eta \right)$. These parameters are namely as Biot numbers $\left({Bi}_{1}, {Bi}_{2}\right)$, Brinkman number $Br$, Eckert number $Ec$, Prandtl number $Pr$, diffusive variable ${\lambda }_{1}$, magnetic parameter $M$, Brownian motion parameter ${N}_{b}$, thermophoresis parameter ${N}_{t}$, radiation parameter ${R}_{d}$, Casson fluid parameter $\beta$, slip parameter $\delta$ and concentration gradient parameter $\chi$.

**Figure 3:**
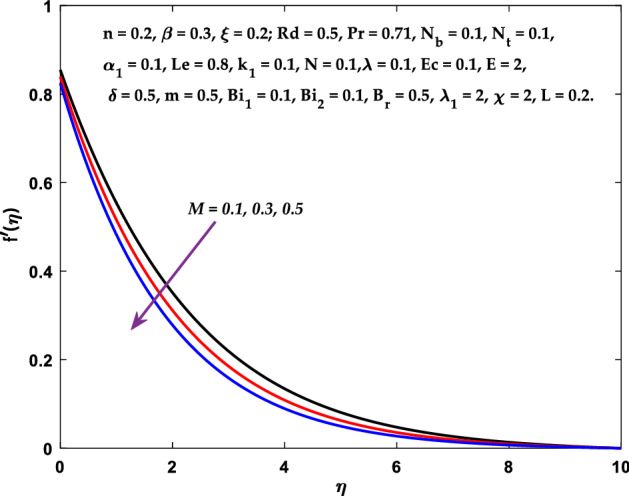
Diagram of $$M$$ v/s $${f}^{^{\prime}}\left(\eta \right).$$

**Figure 4:**
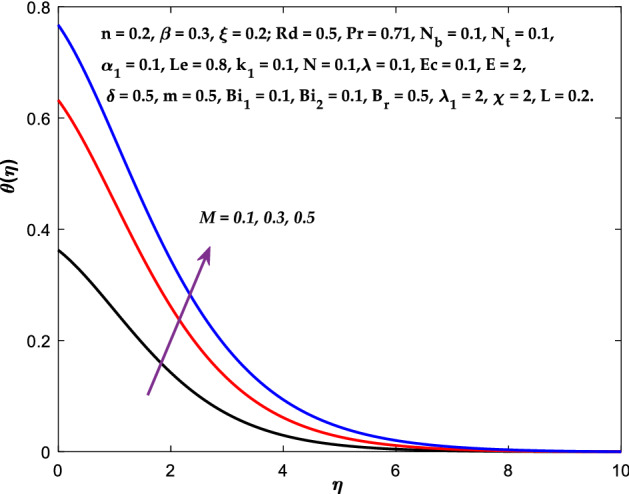
Diagram of $$M$$ v/s $$\theta \left(\eta \right).$$

**Figure 5:**
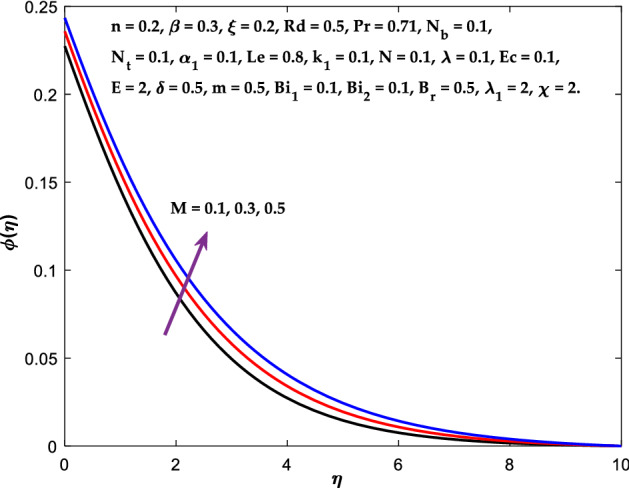
Diagram of $$M$$ v/s $$\phi \left(\eta \right).$$

**Figure 6:**
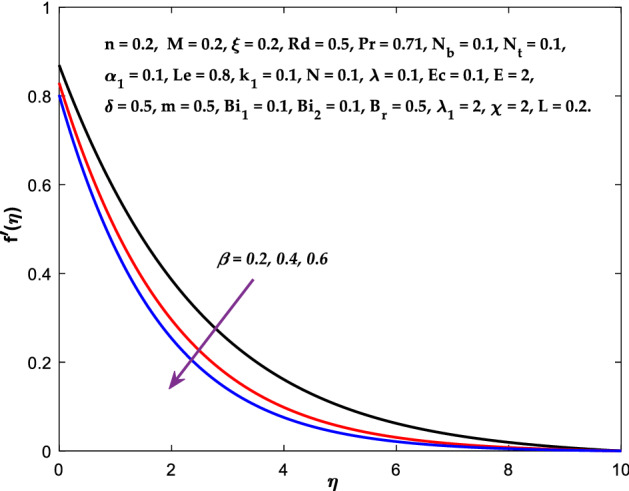
Diagram of $$\beta$$ v/s $$f^{\prime}\left( \eta \right).$$

**Figure 7:**
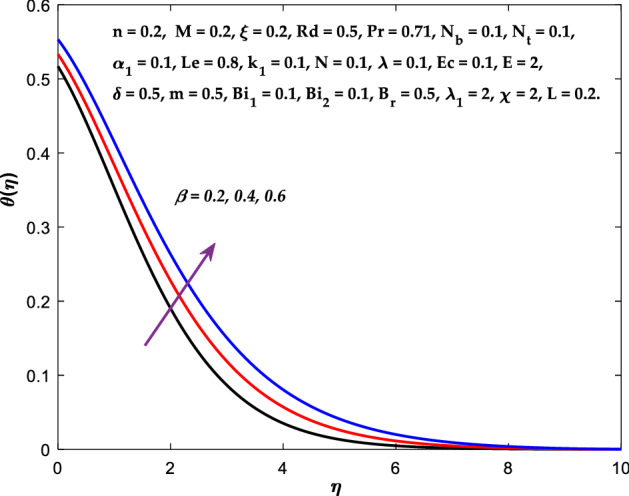
Diagram of $$\beta$$ v/s $$\theta \left(\eta \right).$$

**Figure 8:**
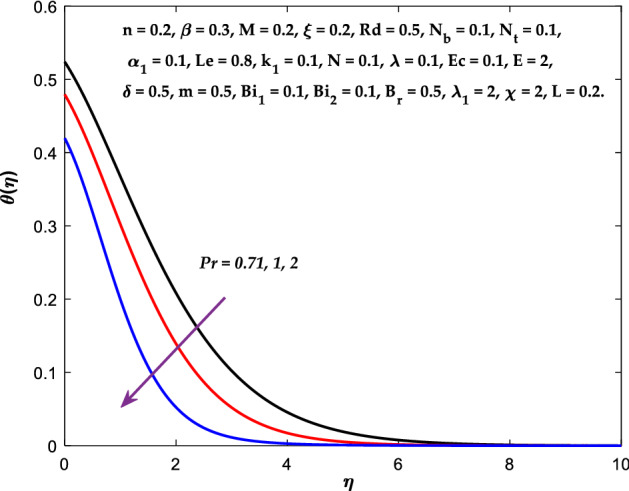
Diagram of $$Pr$$ v/s $$\theta \left(\eta \right).$$

**Figure 9:**
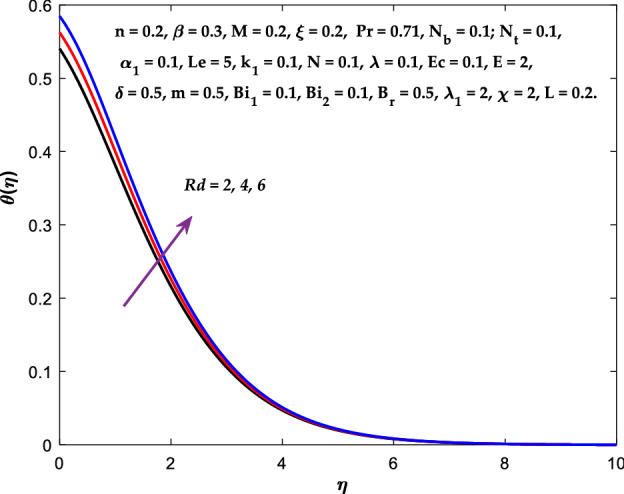
Diagram of $${R}_{d}$$ v/s $$\theta \left(\eta \right).$$

**Figure 10:**
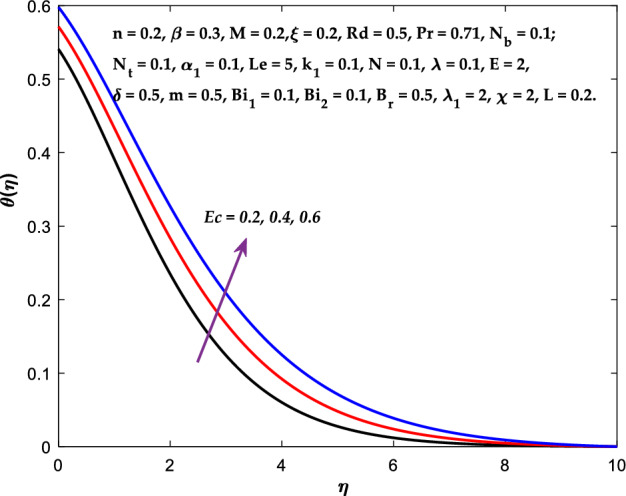
Diagram of $$Ec$$ v/s $$\theta \left(\eta \right).$$

**Figure 11:**
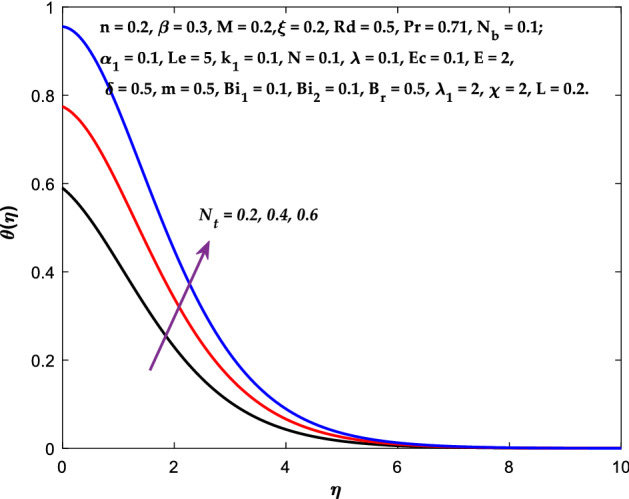
Diagram of $${N}_{t}$$ v/s $$\theta \left(\eta \right).$$

**Figure 12:**
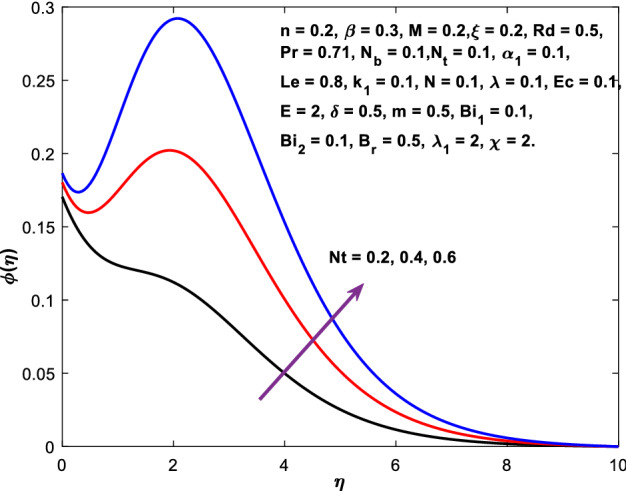
Diagram of $${N}_{t}$$ v/s $$\phi \left(\eta \right).$$

**Figure 13:**
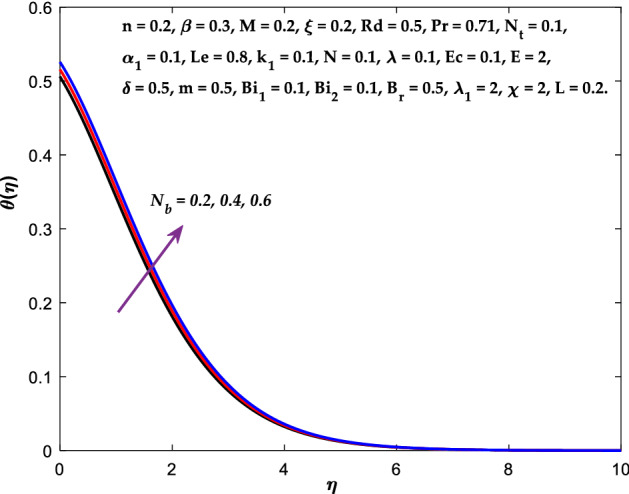
Diagram of $${N}_{b}$$ v/s $$\theta \left(\eta \right).$$

**Figure 14:**
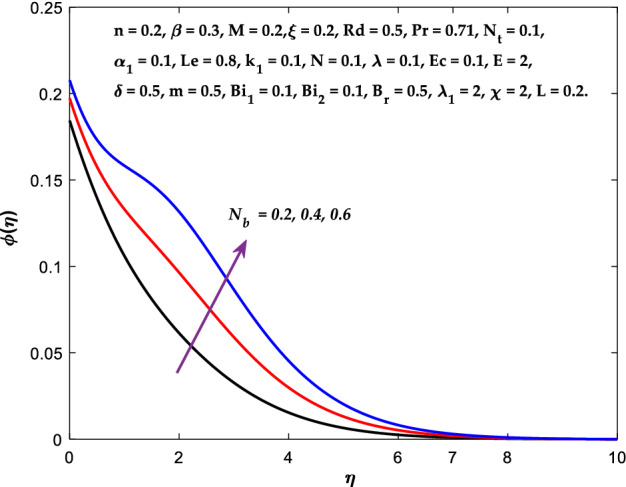
Diagram of $${N}_{b}$$ v/s $$\phi \left(\eta \right).$$

**Figure 15:**
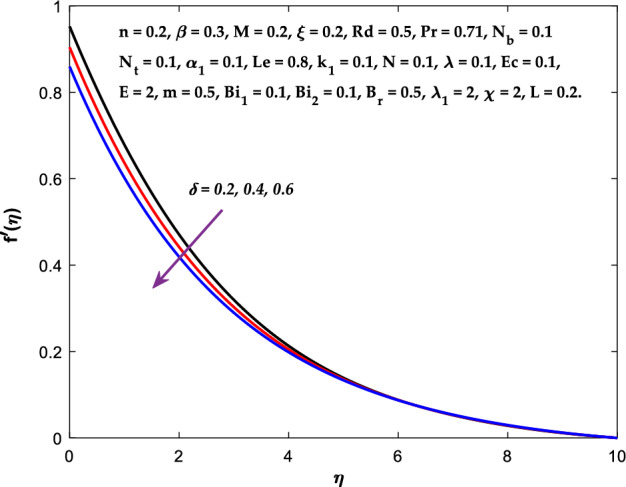
Diagram of $$\delta$$ v/s $$f^{\prime}\left( \eta \right).$$

**Figure 16:**
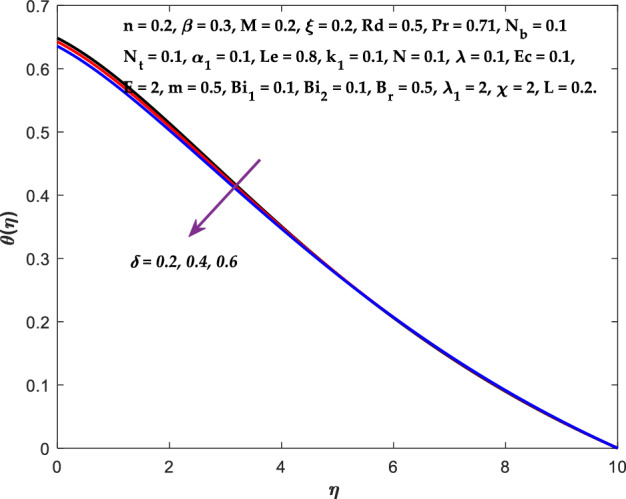
Diagram of $$\delta$$ v/s $$\theta \left(\eta \right).$$

**Figure 17:**
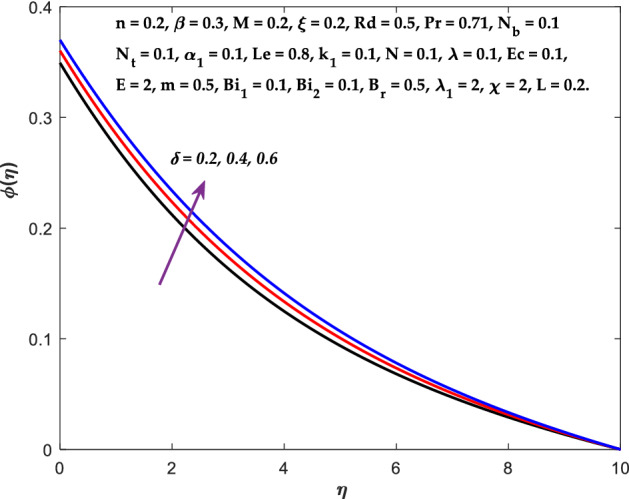
Diagram of $$\delta$$ v/s $$\phi \left(\eta \right).$$

**Figure 18:**
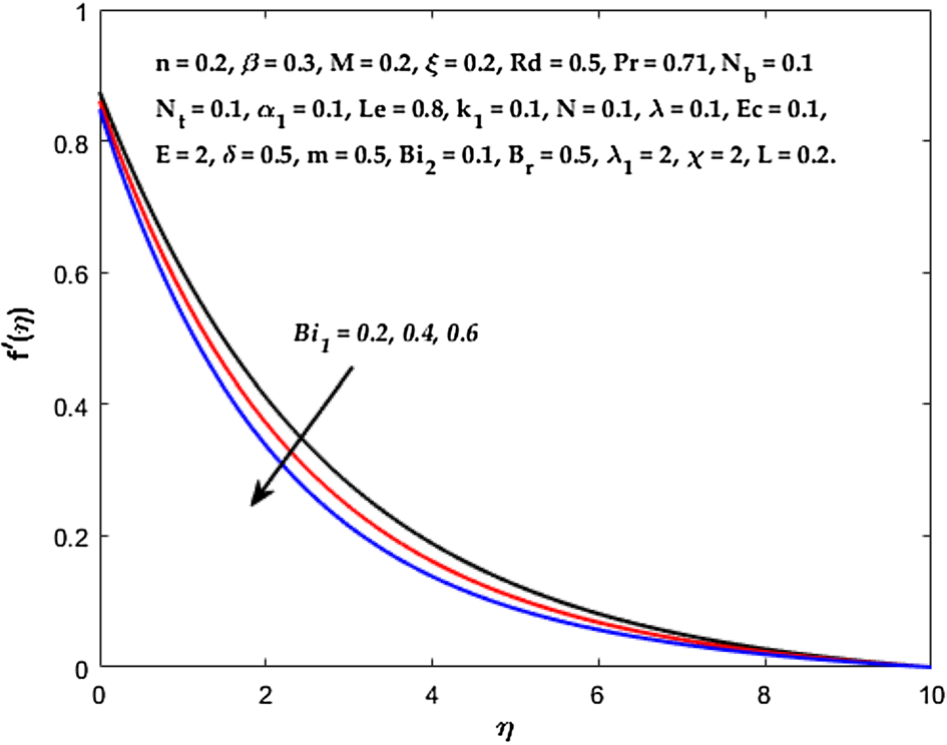
Diagram of $$B{i}_{1}$$ v/s $$f^{\prime}\left( \eta \right).$$

**Figure 19:**
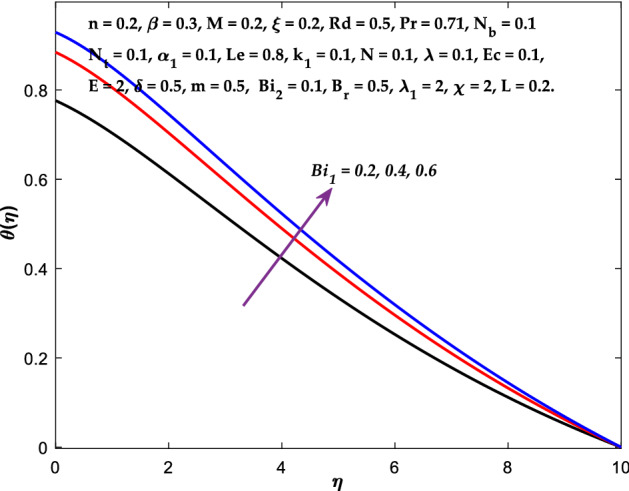
Diagram of $$B{i}_{1}$$ v/s $$\theta \left(\eta \right).$$

**Figure 20:**
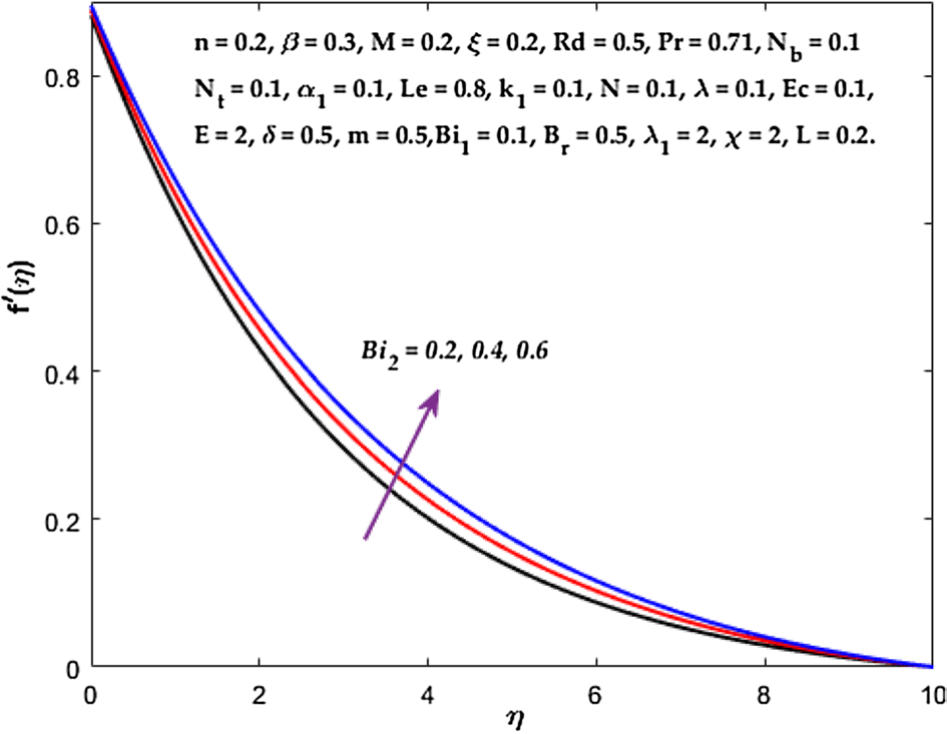
Diagram of $$B{i}_{2}$$ v/s $$f^{\prime}\left( \eta \right).$$

**Figure 21:**
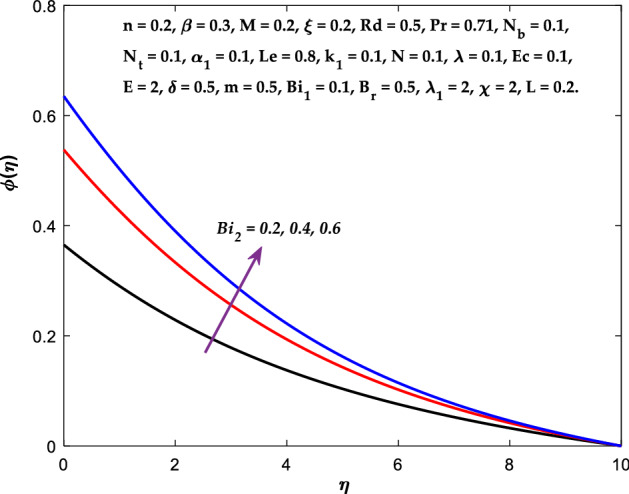
Diagram of $$B{i}_{2}$$ v/s $$\phi \left(\eta \right).$$

Figures 3, 4, 5 are depicted to observe the impact of $M$ on these profiles: $f^{\prime}\left( \eta \right)$, $\theta \left(\eta \right)$ and $\phi \left(\eta \right)$. It has been shown that rising the parameter $M$ leads to a drop in velocity profile while other profiles upsurge (Figs. 4, 5). It is apparent that an enhancement in the parameter $M$ slows the flow while improving other profiles. Raising the magnetic parameter boosts the Lorentz force, which resists the fluid flow. As a result, the Lorentzian force causes an electrically conducting fluid's velocity to decelerate. Figures 6, 7 presents the impression of $\beta$ on $f^{\prime}\left( \eta \right)$ and $\theta \left(\eta \right)$. The velocity reduces, whereas the temperature rises for higher $\beta$. The yield stress drops when the Casson parameter is increased, which lowers the fluid velocity but helps improve the temperature. From a physical perspective, larger $\beta$ values cause a reduction in fluid flow since the flow is under more viscous force. Higher $Pr$ suppresses $\theta \left(\eta \right)$ in Fig. 8. As $Pr$ rises, the thermal conductive falls, and consequently, conduction and even thickness of the thermal boundary layer decays. Therefore, the decrement of thermal boundary layer thickness is the justification of the reduction in temperature for higher $Pr$. The temperature profile in Fig. 9 enhances for larger estimations of the radiation parameter ${R}_{d}$. This consequence can be clarified by the reality that higher estimations of the parameter ${R}_{d}$ for an assumed of ${T}_{\infty }$ leads a decrement in the Rosseland radiative absorptive ${k}_{1}^{*}$. The radiative heat flux divergence $\frac{\partial {q}_{r}}{\partial y}$ enhances as ${k}_{1}^{*}$ decays, increasing the radiative heat transfer rate to the fluid, and causing the fluid temperature to escalate. According to this explanation, the influence of radiation becomes increasingly substantial when ${R}_{d}\to \infty$, and can be ignored as ${R}_{d}\to 0$. Figure 10 exposes the augmented temperature $\theta \left(\eta \right)$ due to higher estimations of the Eckert number $Ec$. An augmentation in $Ec$ leads to a conversion of the kinetic energy to heat energy because of the enhancement in thermal conductivity of the fluid. Consequently, fluid temperature is enhanced. It is well known that heat is produced during viscous dissipation as a result of drag between the fluid particles, and that this additional heat raises the initial fluid temperature. The impact of ${N}_{t}$ on the dimensionless profiles $\theta \left(\eta \right)$ and $\phi \left(\eta \right)$ are delineated in Figs. 11, 12. The temperature increases for higher ${N}_{t}$ as shown in Fig. 11, the concentration observes two different patterns, i.e., decreasing near the wall and increasing away from the wall. Increasing the parameter ${N}_{t}$ generate a temperature gradient, which produces a thermophoretic force between nanoparticles to increase. This force causes more fluid to be heated, which raises the temperature. The same impression is found for nanoparticle concentration by enhancing the parameter ${N}_{t}$ as demonstrated in Fig. 12. A rise in the Brownian motion parameter ${N}_{b}$ causes augmentation in $\theta \left(\eta \right)$ and $\phi \left(\eta \right),$ as shown in Figs. 13, 14. It has been discovered that raising the parameter ${N}_{b}$ the random motion, as well as th collision of the macroscopic fluid particles, escalates, and as a result, temperature increases. Physically, it makes sense because in a nanofluid system, Brownian motion results from the interaction of nanoparticles with the base fluid. The Brownian diffusion displays heat conduction, which is the cause. The sheet surface area for transferring heat is increased by the nanoparticles. The impact of $\delta$ on ${f}^{^{\prime}}\left(\eta \right)$, $\theta \left(\eta \right)$ and $\phi \left(\eta \right)$ are provided in Figs. 15, 16, 17. The profiles $\left({f}^{^{\prime}}\left(\eta \right), \theta \left(\eta \right)\right)$ are decaying for higher estimations of the parameter, whereas the profile $\phi \left(\eta \right)$ enhancing (Fig. 16). With the slip, the flow velocity near the sheet differs from the sheet's stretching velocity. Because the fluid velocity drops as a velocity slip parameter increases, the stretch's sheet tugging can be partially communicated to the fluid. The second reason might be because the fluid's motion is slowed by the increased implications of the velocity slip factor, which causes the fluid to accelerate. The friction forces between the nanofluid and the boundary layer are lessened as the slip velocity parameter is increased. The impact of ${Bi}_{1}$ on ${f}^{^{\prime}}\left(\eta \right)$ and $\theta \left(\eta \right)$ are provided in Figs. 18, 19. It is noticed that upon escalating the parameter ${Bi}_{1}$ leads to a considerable increment in the temperature and decrement in the fluid flow. It is evident that as ${Bi}_{1}$ rises, the heat transfer rate from the warm fluid on the bottom side of the sheet towards the cold fluid on the upper side also rises. As a result, the fluid temperature elevates at the upper side. The increment in ${Bi}_{2}$ leads to the escalation in ${f}^{^{\prime}}\left(\eta \right)$ and $\phi \left(\eta \right)$ distributions, as shown in Figs. 20, 21. The transferred mass will be dispersed throughout the surface by convection and, as a result, increase the nanoparticle concentration. Compared to the constant surface temperature and concentration conditions, the nanofluid with convective boundary conditions is a more relevant model.

**Figure 22:**
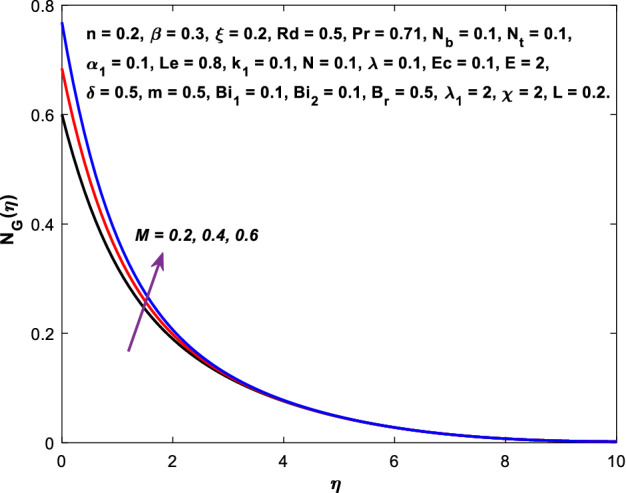
Diagram of $$M$$ v/s $${N}_{G}\left(\eta \right).$$

**Figure 23:**
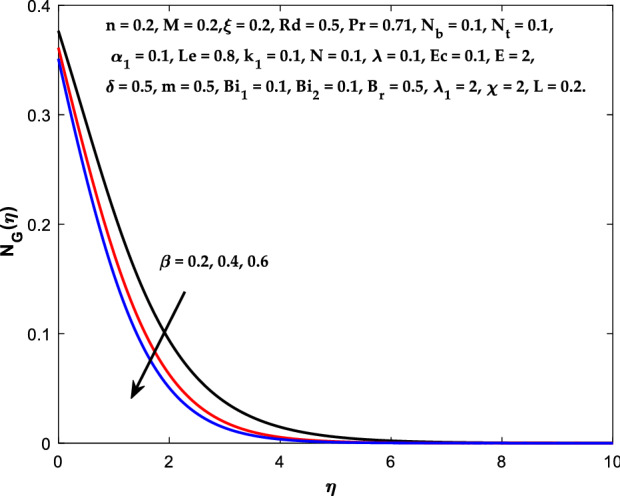
Diagram of $$\beta$$ v/s $${N}_{G}\left(\eta \right).$$

**Figure 24:**
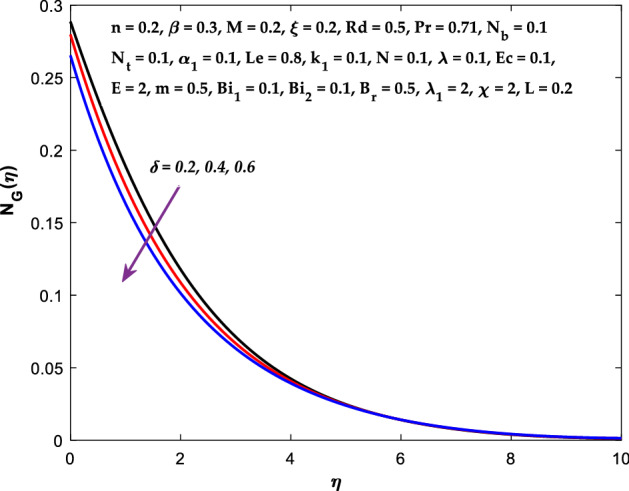
Diagram of $$\delta$$ v/s $${N}_{G}\left(\eta \right).$$

**Figure 25:**
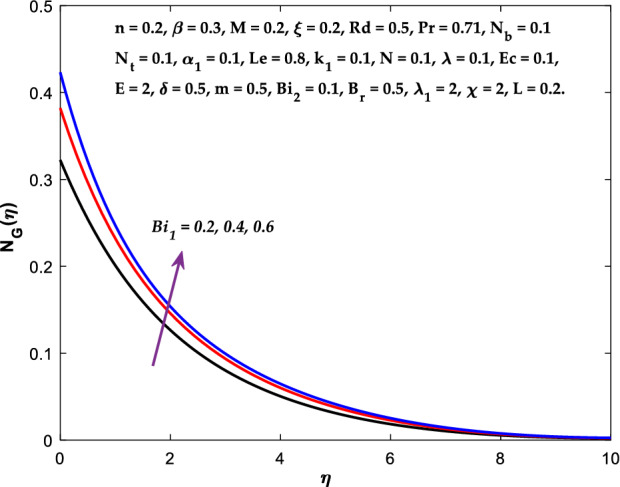
Diagram of $$B{i}_{1}$$ v/s $${N}_{G}\left(\eta \right).$$

**Figure 26:**
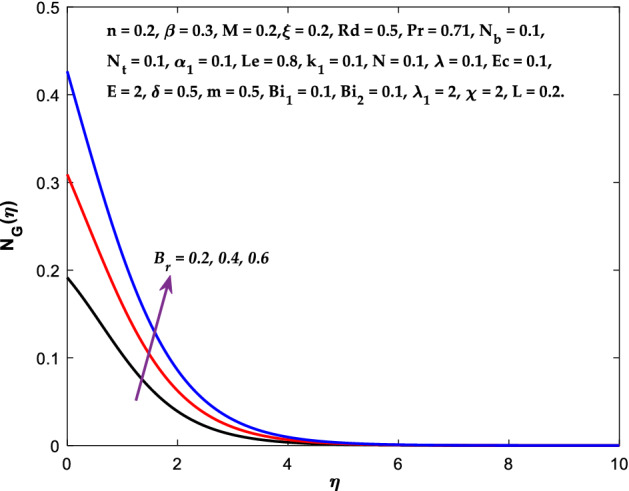
Diagram of $${B}_{r}$$ v/s $${N}_{G}\left(\eta \right).$$

**Figure 27:**
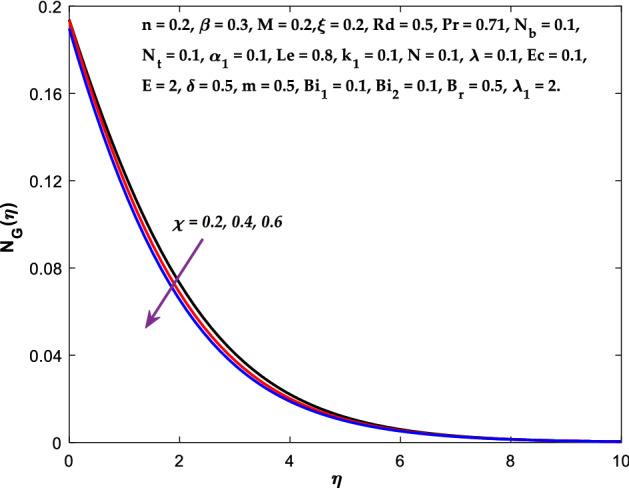
Diagram of $$\chi$$ v/s $${N}_{G}\left(\eta \right).$$

**Figure 28:**
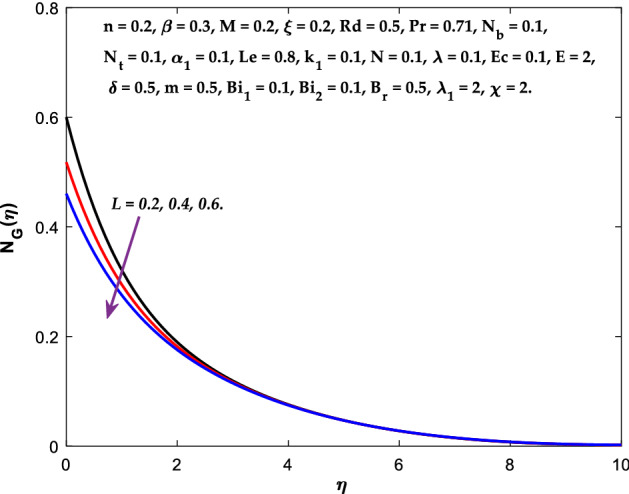
Diagram of $$L$$ v/s $${N}_{G}\left(\eta \right).$$

The impact of $M$ on the entropy production profile ${N}_{G}\left(\eta \right)$ are measured in Fig. 22. The profile ${N}_{G}\left(\eta \right)$ is observed decaying for growing $M$. Physically, the fluid particles motion is resisted by a larger $M$. Consequently, the system produces more disturbance, which increases the creation of entropy. The entropy production ${N}_{G}\left(\eta \right)$ decreases in Fig. 23 for increasing $\beta$. The fluid irreversibility is under control as the Casson parameter increases. Thus the Casson parameter augment the system's obtainable energy as the produced stress drops and fluid viscosity rises. As a result, regulating the Casson fluid parameter can help achieve the goal of limiting entropy creation. Figure 24 shows that the entropy production reduces for rising $\delta$. The impact of ${Bi}_{1}$ on the entropy production ${N}_{G}\left(\eta \right)$ is provided in Fig. 25. This figure shows that as ${Bi}_{1}$ grows, the entropy production also grows. The heat transfer rate enhances as the parameter ${Bi}_{1}$ rises, resulting in higher heat generation and more entropy formation. The variation of the Brinkman number ${B}_{r}$ on entropy production ${N}_{G}\left(\eta \right)$ is captured in Fig. 26. The growth in the parameter ${B}_{r}$ causes escalation in the entropy formation. The parameter ${B}_{r}$ represents the ratio of the heat transfer through conduction to heat production by viscous heating. Therefore, higher ${B}_{r}$ generate more heat in the system, causing a rise in the overall system's disorders. The higher estimations of the concentration gradient parameter $\chi$ and diffusive variable ${\lambda }_{1}$ in Figs. 27, 28 helps to control the entropy production in the system.

Tables 1, 2 are drawn in limiting cases to check the efficiency of the adopted numerical technique. It is observed from these tables that the adopted numerical scheme is highly convergent, and results are correct up to four decimal places with those in literature.

**Table 1:** The numerical results of $\theta \left( 0 \right)\;{\text{and}}\;\theta ^{\prime}(0)$ for increasing $Pr$ when $n=\beta = M =\xi =Rd = {N}_{b} ={N}_{t} = {\alpha }_{1} =Le = {k}_{1} = N = \lambda = Ec = E = \delta = m = {\lambda }_{1} = \chi = {B}_{r} = 0, B{i}_{1} = B{i}_{2} = \infty$.

$Pr$	Salleh et al.^55^	Arifin et al.^56^	Turkyimazoglu^57^	Hasmawani et al.^58^	Present study	Salleh et al.^55^	Present study
	$-\theta (0)$	$-\theta (0)$	$-\theta (0)$	$-\theta (0)$	$-\theta (0)$	$-\theta \mathrm{^{\prime}}(0)$	$-\theta \mathrm{^{\prime}}(0)$
3	6.02577	6.0513	6.05159	6.05159	6.051715	7.02577	7.051715
5	1.76594	1.7604	1.76040	1.76039	1.760392	2.76594	2.760392
7	1.13511	1.1168	1.11681	1.11681	1.116814	2.13511	2.116814
10	0.76531	0.7645	0.76542	0.76452	0.764524	1.76531	1.764524

**Table 2:** The numerical results of $- f\prime \prime (0)$ for increasing $Pr$ when $\beta = M =\xi =Rd = Pr= {N}_{b} ={N}_{t} = {\alpha }_{1} =Le = {k}_{1} = N = \lambda = Ec = E = \delta = m = {\lambda }_{1} = \chi = {B}_{r} = 0, B{i}_{1} = B{i}_{2} = \infty .$

$n$	Cortell^59^	Imran Ullah^60^	Present study
0	0.62755	0.6276	0.627563
0.2	0.76676	0.7668	0.766845
0.5	889,477	0.8896	0.889552
1	1	1	1.000008
3	1.14859	1.1486	1.148601
10	1.23488	1.2349	1.234883
100	1.27677	1.2768	1.276781

The numerical values of the local skin friction coefficients $\sqrt {Re_{x} } Cf_{x}$ , Nusselt number $Nu_{x} /\sqrt {Re_{x} }$ and Sherwood number $Sh_{x} /\sqrt {Re_{x} }$ are calculated in Table 3 for different ranges of $m, M, \beta , \lambda , N$ and $Ec$. In a similary way these quantities are presented in Table 4 for diverse ranges of the parameters $Pr, {R}_{d}, {N}_{t}, {N}_{b}, {Bi}_{1}$ and $\varepsilon$. The $Sh_{x} /\sqrt {Re_{x} }$ values are shown in Table 5 for various ranges of $Le, {k}_{1}$ and ${\alpha }_{1}$.

**Table 3:** The values of $\sqrt {Re_{x} } Cf_{x}$*, *$Nu_{x} /\sqrt {Re_{x} }$*,* and $Sh_{x} /\sqrt {Re_{x} }$*,* for diverse values of $m , M, \beta ,$ $\lambda , N$ and $Ec,$ other values are $n = 0.2, \beta = 0.3, M = 0.2,\xi = 0.2, Rd = 0.5, Pr = 0.71, {N}_{b} = 0.1, {N}_{t} = 0.1, {\alpha }_{1} = 0.1, Le = 0.8, {k}_{1} = 0.1 ,E = 2, \delta = 0.5, m = 0.5, B{i}_{1} = 0.1, B{i}_{2} = 0.1, {B}_{r} = 0.5, {\lambda }_{1} = 2, \chi = 2.$

$m$	$M$	$\beta$	$\lambda$	$N$	$Ec$	$\sqrt{R{e}_{x}}C{f}_{x}$	$N{u}_{x}/\sqrt{R{e}_{x}}$	$S{h}_{x}/\sqrt{R{e}_{x}}$
0.2	0.2	0.3	0.1	0.1	0.1	−1.34283	−0.03958	0.086252
0.4						−1.480704	−0.03817	0.131768
0.6						−1.594115	−0.03577	0.168437
0.2	0.4					−1.338124	−0.03915	0.086245
	0.6					−1.333419	−0.03873	0.086238
	0.2	0.5				−0.929407	−0.04016	0.082269
		0.7				−0.75227	−0.04076	0.080563
		0.3	0.3			−2.006734	0.009245	0.077351
			0.5			−2.3163	0.023558	0.072928
			0.1	0.3		−1.338138	−0.27206	0.103882
				0.5		−1.336041	−0.48115	0.120066
				0.1	0.3	−1.34574	−0.03942	0.120356
					0.5	−1.346853	−0.03923	0.137289

**Table 4:** The values of $\sqrt {Re_{x} } Cf_{x}$*, *$Nu_{x} /\sqrt {Re_{x} }$*,* and $Sh_{x} /\sqrt {Re_{x} }$ *,*for diverse values of $Pr, Rd, Nt, Nb,B{i}_{1}$ and $\varepsilon$ when $n = 0.2, \beta = 0.3, M = 0.2,{R}_{d} = 0.5, {\alpha }_{1} = 0.1, Le = 0.8, {k}_{1} = 0.1, N = 0.1, \lambda = 0.1, Ec = 0.1,E = 2, \delta = 0.5, m = 0.5, B{i}_{2} = 0.1, {B}_{r} = 0.5, {\lambda }_{1} = 2, \chi = 2.$

$Pr$	${R}_{d}$	$Nt$	$Nb$	$B{i}_{1}$	$\varepsilon$	$\sqrt{R{e}_{x}} C{f}_{x}$	$N{u}_{x}/\sqrt{R{e}_{x}}$	$S{h}_{x}/\sqrt{R{e}_{x}}$
0.71	0.1	0.1	0.1	0.1	0.2	−1.349513	0.059277	0.076903
1						−1.34935	0.058238	0.077401
3						−1.348487	0.051409	0.082968
0.71	0.3					−1.346175	0.019985	0.081586
	0.5					−1.34283	−0.03958	0.086252
		0.3				−1.343719	0.017321	0.081255
		0.5				−1.334799	−0.04382	0.087486
			0.3			−1.349082	0.059913	0.07681
			0.5			−1.348809	0.060489	0.076733
				0.3		−1.349513	0.059277	0.076903
				0.5		−1.349513	0.059277	0.076903
					0.4	−1.350426	0.059268	0.085958
					0.6	−1.351035	0.059269	0.092205

**Table 5:** The values of $\frac{{Nu_{x} }}{{\sqrt {Re_{x} } }}$*,* for increasing $Le, {K}_{1}$ and ${\alpha }_{1}$ when $n = 0.2,\beta = 0.3, M=0.2, \varepsilon = 0.2,Rd = 0.5, Pr= 0.71,{N}_{b}= 0.1 {N}_{t} = 0.1,N = 0.1,\lambda = 0.1,Ec = 0.1, E=2,$ $\delta = 0.5,m = 0.5,B{i}_{1}= 0.1,B{i}_{2}= 0.1,{B}_{r} = 0.5,{\lambda }_{1} = 2,\chi = 2.$

$Le$	${k}_{1}$	${\alpha }_{1}$	$S{h}_{x}/\sqrt{R{e}_{x}}$
0.4	0.1	0.1	0.076369
0.6			0.076656
0.8			0.076903
	0.3		0.07797
	0.5		0.079098
	0.1	0.3	0.076203
		0.5	0.074869

## Conclusions

A theoretical entropy production analysis is carried out in mixed convective electrically conducting Casson type nanofluid flow subjected to the factors of thermal radiation, viscous dissipation, and joule heating, heat generation/absorption, and activation energy. In addition, the Casson nanofluid flow also has been bounded by the slip and convective conditions. The simulations are performed numerically, and thus the following conclusion is drawn from the present analysis:

- The fluid velocity is effectively controlled through the parameters $M, \beta ,$ and $\delta$.
- The fluid temperature gets enhanced for the parameters $M, \beta , {R}_{d}, Ec, {N}_{t}, {N}_{b}, {Bi}_{1}$ but it decays for the parameters $Pr, \delta$
- The concentration of the nanoparticles boosts for the parameters $M, {N}_{t}, {N}_{b}, \delta , {Bi}_{2}$ whereas it reduces for the parameters.
- The enhancement in the parameters $M,{Bi}_{1}, {B}_{r}$ leads to an increment in the entropy generation, whereas the parameters $\beta , \delta , \chi ,$ and $L$ help to minimize the entropy production.

For future recommendations, this model can be extended for the different types of non-Newtonian fluid such as Maxwell, Carreau, etc. Besides, the extension of this model can be performed by substituting the differed stretching or shrinking sheet instead of the flat one, such as cylinder, cone or wedge. The bvp4c MATLAB method could be applied to a variety of physical and technical challenges in the future^61–77^.

## Data Availability

All data generated or analysed during this study are included in this published article.

## Roman letters

$$a$$: Reference length (m)
$${B}_{r}$$: Brinkman number
${B}_{0}$: Strength of magnetic field (kgs^-2^ A^-1^)
$${Bi}_{1}, {Bi}_{2}$$: Biot numbers
$${C}_{W}$$: Wall Concentration
$${C}_{\infty }$$: Ambient concentration
${C}_{{f}_{x}}$: Skin friction coefficient
${c}_{f}$: Specific heat of fluid (J/(K.kg))
${c}_{p}$: Specific heat of nanoparticles
${D}_{B}$: Brownian diffusion coefficient (kg m^−1^ s^−1^)
${D}_{T}$: Thermophoretic diffusion coefficient (m^2^ s^−1^)
$E$: Activation energy parameter
$${E}_{c}$$: Eckert number
$Gr$: Thermal Grashof number
$g$: Gravitational force due to acceleration
${h}_{f}$: Convective heat transfer (W/m^2^·K)
${h}_{s}$: Convective mass transfer
$k$: Thermal conductivity of the fluid (W/m·K)
$${k}_{1}$$: Reaction rate
${k}_{1}^{*}$: Mean absorption coefficient
$${L}_{e}$$: Lewis number
$$M$$: Magnetic parameter
$N$: Buoyancy forces ratio
$${N}_{t}$$: Thermophoresis parameter
${N}_{b}$: Brownian motion parameter
${N}_{{u}_{x}}$: Local Nusselt number
$${P}_{r}$$: Prandtl number
$Q$: Heat generation/absorption coefficient
${q}_{r}$: Radiative heat flux
${q}_{w}$: Wall heat flux
${q}_{s}$: Wall mass flux
$${R}_{d}$$: Radiation parameter
$R{e}_{x}$: Local Reynold number
$S{h}_{x}$: Local Sherwood number
$$T$$: Fluid Temperature (K)
${T}_{f}$: Convective Fluid temperature
${T}_{\infty }$: Fluid ambient temperature
$$u, v$$: Velocity components (m/s)
${u}_{w}$: Stretching sheet velocity (m/s)
$$x,y$$: Coordinate axis

## Greek letters

$${\alpha }_{f}$$: Thermal diffusivity
$${\alpha }_{1}$$: Temperature gradient
$\beta$: Casson fluid parameter
${\beta }_{T}$: Volumetric coefficient of thermal expansion
$$\delta$$: Slip parameter
$$\eta$$: Similarity variable
$\varepsilon$: Heat generation/absorption parameter
$\lambda$: Mixed convection parameter
${\mu }_{f}$: Dynamic viscosity of fluid (kg/ms^3^)
$$\nu$$: Kinematic viscosity (m^2^/s)
${\rho }_{f}$: Fluid Density (kg/m^3^)
${\rho }_{p}$: Density of nanoparticles
$\phi$: Dimensionless nanoparticle concentration
$$\psi$$: Stream function
$$\sigma$$: Electrical conductivity
${\sigma }^{*}$: Stefan-Boltzmann constant
$\tau$: Ratio of heat capacities
${\tau }_{w}$: Wall shear stress
$$\theta$$: Dimensionless temperature

## Subscripts

$\infty$: Condition at free stream
$w$: Condition at wall
