# Author Correction: Partial differential equations modeling of thermal transportation in Casson nanofluid flow with arrhenius activation energy and irreversibility processes

**DOI:** 10.1038/s41598-023-28885-6

**Published:** 2023-01-31

**Authors:** Khalid Fanoukh Al Oweidi, Wasim Jamshed, B. Shankar Goud, Imran Ullah, Usman, Siti Suzilliana Putri Mohamed Isa, Sayed M. El Din, Kamel Guedri, Refed Adnan Jaleel

**Affiliations:** 1 Department of Water Resources Management Engineering, College of Engineering, Al-Qasim Green University, Babylon, Iraq; 2 Department of Mathematics, Capital University of Science and Technology (CUST), Islamabad, 44000 Pakistan; 3 Department of Mathematics, JNTUH University College of Engineering Hyderabad, Kukatpally, Hyderabad, Telangana 500085 India; 4 College of Civil Engineering, National University of Sciences and Technology, Islamabad, 44000 Pakistan; 5 Department of Computer Science, National University of Sciences and Technology, Balochistan Campus (NBC), Quetta, 87300 Pakistan; 6 Institute for Mathematical Research, Universiti Putra Malaysia (UPM), 43400 Serdang, Selangor Darul Ehsan Malaysia; 7 Centre of Foundation Studies for Agricultural Science, Universiti Putra Malaysia (UPM), 43400 Serdang, Selangor Malaysia; 8 Faculty of Engineering, Center of Research, Future University in Egypt, New Cairo, 11835 Egypt; 9 Mechanical Engineering Department, College of Engineering and Islamic Architecture, Umm Al-Qura University, P. O. Box 5555, Makkah, 21955 Saudi Arabia; 10 Department of Information and Communication Enginèering, Al-Nahrain University, Baghdad, Iraq

Correction to: *Scientific Reports* 10.1038/s41598-022-25010-x, published online 29 November 2022

In the original version of this Article B. Shankar Goud was incorrectly affiliated with ‘College of Civil Engineering, National University of Sciences and Technology, Islamabad 44000, Pakistan’. The correct affiliation is listed below.

Department of Mathematics, JNTUH University College of Engineering Hyderabad, Kukatpally, Hyderabad, Telangana 500085, India.

Additionally, Imran Ullah was incorrectly affiliated with ‘Department of Mathematics, JNTUH University College of Engineering Hyderabad, Kukatpally, Hyderabad, Telangana 500085, India’. The correct affiliation is listed below.

College of Civil Engineering, National University of Sciences and Technology, Islamabad 44000, Pakistan.

The original Article has been corrected.

